# Progressive Failure Analysis of Laminates with Embedded Wrinkle Defects Based on an Elastoplastic Damage Model

**DOI:** 10.3390/ma13102422

**Published:** 2020-05-25

**Authors:** Zhi Hua Ning, Guan Liang Huo, Ren Huai Liu, Wei Lin Wu, Jia Ming Xie

**Affiliations:** MOE Key Laboratory of Disaster Forecast and Control in Engineering, School of Mechanics and Construction Engineering, Jinan University, Guangzhou 510632, China; guanlianghuo@stu2017.jnu.edu.cn (G.L.H.); lrh@jnu.edu.cn (R.H.L.); wwl1122@stu2016.jnu.edu.cn (W.L.W.); xiejiaming123@stu2016.jnu.edu.cn (J.M.X.)

**Keywords:** elastoplastic damage model, wrinkle defect, progressive failure analysis, fiber kinking, fracture plane

## Abstract

Out-of-plane wrinkling has a significant influence on the mechanical performance of composite laminates. Numerical simulations were conducted to investigate the progressive failure behavior of fiber-reinforced composite laminates with out-of-plane wrinkle defects subjected to axial compression. To describe the material degradation, a three-dimensional elastoplastic damage model with four damage modes (i.e., fiber tensile failure, matrix failure, fiber kinking/splitting, and delamination) was developed based on the LaRC05 criterion. To improve the computational efficiency in searching for the fracture angle in the matrix failure analysis, a high-efficiency and robust modified algorithm that combines the golden section search method with an inverse interpolation based on an existing study is proposed. The elastoplastic damage model was implemented in the finite-element code Abaqus using a user-defined material subroutine in Abaqus/Explicit. The model was applied to the progressive failure analysis of IM7/8552 composite laminates with out-of-plane wrinkles subjected to axial compressive loading. The numerical results showed that the compressive strength prediction obtained by the elastoplastic damage model is more accurate than that derived with an elastic damage model. The present model can describe the nonlinearity of the laminate during the damage evolution and determine the correct damage locations, which are in good agreement with experimental observations. Furthermore, it was discovered that the plasticity effects should not be neglected in laminates with low wrinkle levels.

## 1. Introduction

Fiber waviness is a type of manufacturing defect that occurs mostly during filament winding. Ply level out-of-plane waviness can result in severe degradation of mechanical properties, in particular, such as the compressive strength of composites. Hsiao and Daniel [1] conducted theoretical and experimental studies on unidirectional composites with out-of-plane wrinkles under compressive loading. They discovered that the stiffness and strength of the laminates decreases significantly with increasing fiber waviness. A similar study was conducted by Davidson and Waas [2], who found that for thick unidirectional carbon fiber polymer matrix composites, there exists a fiber misalignment angle at which the compressive strength is the global minimum. In a subsequent work, Davidson and Waas [3] developed a fiber waviness tolerance and criticality assessment framework employing surrogate modeling and Monte Carlo methods to predict the compressive strength and failure mode of a composite structure with fiber waviness. Adams et al. [4,5] investigated the effect of fiber wrinkles on the compressive strength of symmetric cross-ply laminates. According to their results, the compressive strength of those laminates was up to 36% lower than that of pristine laminates. Furthermore, various studies have shown that composites exhibit significant nonlinear behaviors before the final collapse of their structures, especially for laminates with wrinkles. Wisnom and Atkinson [6] performed finite-element (FE) analyses and experiments on T800/924 unidirectional laminates. Their results showed that fiber misalignments could induce shear nonlinearities. Chun et al. [7] discovered experimentally that DMS 2224 carbon/epoxy composite laminates with wrinkles exhibit both material and geometric nonlinearities under tension and compression. In addition, Makeev et al. [8] observed a nonlinear shear behavior in IM7/8552 laminates with fiber wrinkles using digital image correlation, and they proposed a nonlinear shear stress–strain relation obtained from numerical simulations. Davidson and Waas [9] used an odd polynomial series fitting obtained from experimental results to model the nonlinear shear response of the matrix in fiber-reinforced composites. Mukhopadhyay et al. [10] captured the progressive damage in IM7/8552 laminates with wrinkles using a high-speed video camera during compression experiments, and then, adopted a nonlinear shear stress–strain relation to describe the material behavior in a subsequent numerical analysis. However, the above studies focused only on shear nonlinearity, and disregarded plasticity and nonlinearity in the transverse direction.

In many progressive damage models developed for composite laminates, plasticity has been introduced to describe material nonlinearity. In a microscale, Prabhakar and Waas [11,12,13], Sun et al. [14], and Yuan et al. [15] applied a micromechanics model to predict the compressive failure behavior of unidirectional fiber-reinforced laminated composites using plasticity to approximate the nonlinearity of matrices. At the ply level, Lemanski et al. [16] presented a perfectly plastic model based on the Hill yield criterion and the kinematic hardening rule to simulate the nonlinear behavior of AS4/8552 composite laminates fabricated by Wang et al. [17], although only delamination damage was considered in their analysis. Wang et al. [18,19,20] performed off-axis tension and compression tests of IM600/Q133 unidirectional laminates and discovered that a material performs differently under tension and compression. Therefore, they proposed an elastoplastic constitutive model with distinguished tension and compression performances as an improvement on the Hill yield criterion. Xue et al. [21,22] applied this model to analyze the progressive elastoplastic failure behavior of IM600/Q133 and IM7/8552 composite laminates. Chen et al. [23] developed a combined elastoplastic damage model that accounted for plasticity on both the transverse and in-plane shear directions; however, the model could only be applied for two-dimensional damage analysis. According to an experiment, in which composite laminates were subjected to traverse compression [24,25], the fracture plane is not parallel to the loading direction. Moreover, fibers exhibit kinking or splitting failure under axial compression [26,27]. Therefore, existing damage models cannot be applied for the compression failure analysis of laminates considering plasticity effects.

To the best of our knowledge, no studies have been conducted on the longitudinal compression failure of multi-ply laminates with wrinkles that consider plastic damage in the matrix. Therefore, we extended the elastoplastic damage model described in [23] to a three-dimensional (3D) damage analysis and conducted a progressive failure simulation of composite laminates with out-of-plane wrinkle defects subjected to axial compression. To rapidly search for the fracture angle in the matrix failure analysis, we propose a highly accurate approach that combines the golden section search method and an inverse interpolation. We implemented the damage model based on the LaRC05 criterion [28] in a modified algorithm using a user-defined material subroutine in Abaqus/Explicit (VUMAT). To evaluate the effectiveness of the proposed method, we compared the predicted results with experimental observations [10].

The contributions of this study are as follows: (1) We conducted a progressive failure analysis of multidirectional fiber-reinforced polymer laminates with embedded wrinkle defects based on an elastoplastic damage model. (2) We demonstrated the nonlinearity of the laminate during damage evolution and correctly determined the damage location through numerical simulations.

The remainder of the paper is organized as follows: Section 2 describes the elastoplastic damage model adopted in the present study. In Section 3, an approach is proposed to determine the orientation of the fracture plane in the matrix failure analysis. The implementation of the elastoplastic model using the user-defined subroutine VUMAT is presented in Section 4. Section 5 shows numerical simulation examples and discusses the predictions by comparing them with test results [10]. Finally, conclusions are presented in Section 6.

## 2. Elastoplastic Damage Model

### 2.1. Stress-Strain Relationships

For composite materials exhibiting a plasticity response, the total strain tensor **ε** is expressed as the sum of the elastic and plastic strain parts, **ε^e^** and **ε^p^**, respectively, as follows:

(1) $$\varepsilon =\varepsilon^{e}+\varepsilon^{p}$$

According to damage mechanics theories, the Cauchy nominal stress tensor **σ** and the effective stress tensor $\bar{\sigma }$ obey the following relationship:

(2) $$\bar{\sigma }=M\left(d\right)\sigma$$

where $\bar{\sigma }=\left[{\bar{\sigma }}_{11}\;{\bar{\sigma }}_{22}\;{\bar{\sigma }}_{33}\;{\bar{\sigma }}_{23}\;{\bar{\sigma }}_{31}\;{\bar{\sigma }}_{12}\right]$ and $\sigma =\left[\sigma_{11}\;\sigma_{22}\;\sigma_{33}\;\sigma_{23}\;\sigma_{31}\;\sigma_{12}\right]$ for 3D problems. **M**(*d*) = diag[1/(1-*d*_1_) 1/(1-*d*_2_) 1/(1-*d*_2_) 1/(1-*d*_3_) 1/(1-*d*_3_) 1/(1-*d*_2_)], where *d* represents the damage variable and *d*_1_, *d*_2_, and *d*_3_ denote the damage in the fiber and in the transverse and shear directions, respectively. To ensure the irreversibility of the damage, *d*_1_, *d*_2_, and *d*_3_ are expressed as:

(3) $$d_{1}=\max(d_{ft},d_{kink}\;\mathrm{or}\;d_{split}),\;d_{3}=1-\left(1-d_{1}\right)\left(1-d_{2}\right)d_{2}=\max(d_{mt},d_{mc})$$

where *d_ft_* is the damage caused by tension in the fiber direction; *d_kink_* and *d_split_* denote the fiber kinking and splitting, respectively, caused by compression in the fiber direction; *d_mt_* represents both the damage caused by tension in the matrix and the degradation of the interface between the fibers and the matrix due to decohesion; and *d_mc_* denotes the damage caused by compression in the matrix.

The relationship between stress and strain for undamaged orthotropic anisotropy composites is as follows:

(4) $$\bar{\sigma }=C_{0}^{}\varepsilon^{e}$$

where **C**_0_ is the stiffness tensor of the undamaged unidirectional laminated composite. 

By substituting Equation (4) into Equation (2), the relationship between the elastic strain tensor **ε^e^** and the Cauchy nominal stress tensor can be expressed as follows:

(5) $$\varepsilon^{e}=C_{0}^{-1}M\left(d\right)\sigma =S_{0}M\left(d\right)\sigma$$

where **S**_0_ is the undamaged flexibility tensor.

The stress–strain relationship for damaged composite materials can be expressed in the form:

(6) $$\varepsilon^{e}=S_{d}\sigma$$

where **S**_d_ is the damage flexibility tensor.

By comparing Equations (5) and (6), the relationship between **S**_0_ and **S**_d_ can be expressed as follows:

(7) $$S_{d}=S_{0}M\left(d\right)$$

According to Matzenmiller’s assumption [29], the compliance tensor of the damaged lamina can be obtained by adjusting the Poisson ratios. In the present study, the compliance tensor was degraded similar to [29]. Therefore, the following form of **S**_d_ was adopted:

(8) $$S_{d}^{}=\left[\begin{array}{cccccc} \frac{1}{\left(1-d_{1}\right)E_{11}} & \frac{-\upsilon_{12}}{E_{11}} & \frac{-\upsilon_{13}}{E_{11}} & & & \\ \frac{-\upsilon_{12}}{E_{11}} & \frac{1}{\left(1-d_{2}\right)E_{22}} & \frac{-\upsilon_{23}}{E_{22}} & & & \\ \frac{-\upsilon_{13}}{E_{11}} & \frac{-\upsilon_{23}}{E_{33}} & \frac{1}{\left(1-d_{2}\right)E_{33}} & & & \\ & & & \frac{1}{\left(1-d_{3}\right)G_{23}} & & \\ & & & & \frac{1}{\left(1-d_{3}\right)G_{13}} & \\ & & & & & \frac{1}{\left(1-d_{2}\right)G_{12}} \end{array}\right]$$

### 2.2. Plastic Model

The plastic yield function can be expressed in terms of effective stresses as follows [23]:

(9) $$F\left(\bar{\sigma },{\tilde{\varepsilon }}^{p}\right)=F^{p}\left(\bar{\sigma }\right)-\kappa \left({\tilde{\varepsilon }}^{p}\right)$$

where *F^p^* is the plastic potential and *k* is the hardening parameter. The power law proposed by Sun and Chen [30] is expressed as follows:

(10) $${\tilde{\varepsilon }}^{p}=A{\tilde{\sigma }}^{n}$$

where *A* and *n* are coefficients that can be determined using an approach based on the linear regression analysis of off-axis tensile tests performed on unidirectional composite laminate specimens. $\tilde{\sigma }$ and ${\tilde{\varepsilon }}^{p}$ are the equivalent stress and the equivalent plastic strain, respectively.

For simplicity, Chen et al. [23] converted the isotropic hardening law to an equivalent form in which the equivalent plastic strain ${\tilde{\varepsilon }}^{p}$ is used as an internal variable:

(11) $$\kappa \left({\tilde{\varepsilon }}^{p}\right)=\beta {\left({\tilde{\varepsilon }}^{p}\right)}^{m}$$

where the coefficients *β* and *m* are related to *A* and *n*, respectively, through the relationships *β* = *A*^−1/*n*^ and *m* = 1/*n*.

Here, the 3D general plastic potential proposed by Sun and Chen [30] is employed. Hence, *F^p^* can be rewritten as:

(12) $$F^{p}=\tilde{\sigma }=\sqrt{\frac{3}{2}\left[{\left({\bar{\sigma }}_{22}-{\bar{\sigma }}_{33}\right)}^{2}+4{\bar{\sigma }}_{23}^{2}+2a_{66}\left({\bar{\sigma }}_{31}^{2}+{\bar{\sigma }}_{12}^{2}\right)\right]}$$

where *a*_66_ is a material parameter that describes the level of plastic deformation developed under shear loading compared with that under transverse loading and ${\bar{\sigma }}_{ij}$ represents the effective stress component.

Assuming the associated plastic-flow rule and the associated hardening rule for composite materials, the plastic strain rate $d\varepsilon_{ij}^{p}$ can be expressed as:

(13) $$d\varepsilon_{ij}^{p}=\lambda \frac{\partial F}{\partial {\bar{\sigma }}_{ij}}$$

where *λ* is a plastic consistency parameter. 

The plastic work per unit volume *dW^p^* is defined as:

(14) $$dW^{p}={\bar{\sigma }}_{ij}d\varepsilon_{ij}^{p}=\tilde{\sigma }d{\tilde{\varepsilon }}^{p}$$

By substituting Equations (12) and (13) into Equation (14), the equivalent strain rate is expressed as follows:

(15) $$d{\tilde{\varepsilon }}^{p}=\lambda$$

### 2.3. Damage Model

In general, the damage modes in composite structures include fiber failure, matrix cracking, and delamination. Fiber failure and matrix cracking occur in the plane and can be further categorized into tensile and compressive modes. Pinho [28] classified the fiber failure caused by compression into fiber splitting and fiber kinking.

#### 2.3.1. Fiber Tensile Failure

Typically, fiber failure occurs when the longitudinal stress reaches the longitudinal tensile strength. Therefore, the maximum stress criterion is considered:

(16) $$f_{ft}=\frac{{\bar{\sigma }}_{11}}{X_{T}}=1,\;{\bar{\sigma }}_{11}>0$$

where ${\bar{\sigma }}_{11}$ is the normal stress in the fiber direction; *X_T_* is the axial tensile strength of the composite; and *f_ft_* is the exposure factor corresponding to the tension-induced fiber failure.

#### 2.3.2. Matrix Failure

**Figure 1:**
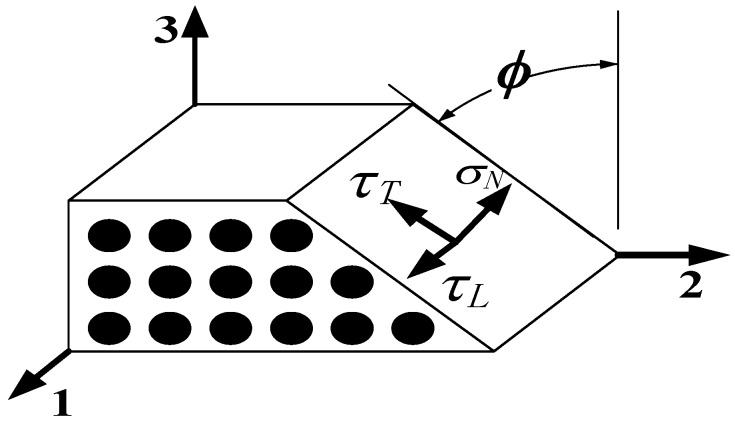
Traction components in the fracture plane, based on [31].

According to experimental observations [24,25], a fracture plane appears in the longitudinal direction of fibers under a transverse stress or/and an in-plane stress acts. Matrix failure occurs in the fracture plane. Puck introduced a criterion using stress components in the fracture plane. Pinho [28] adopted the advantages of the Puck criterion and improved it in the second World-Wide Failure Exercise (WWFE-II). The stress components on the fracture plane, as shown in Figure 1, can be obtained through a transformation of coordinates using the following formulas:

(17) $$\begin{array}{l} \sigma_{N}={\bar{\sigma }}_{22}\cos^{2}\phi +{\bar{\sigma }}_{33}\sin^{2}\phi +2{\bar{\sigma }}_{23}\sin\phi \cos\phi \\ \tau_{T}=\left({\bar{\sigma }}_{33}-{\bar{\sigma }}_{22}\right)\sin\phi \cos\phi +{\bar{\sigma }}_{23}\left(\cos^{2}\phi -\sin^{2}\phi \right)\\ \tau_{L}={\bar{\sigma }}_{31}\sin\phi +{\bar{\sigma }}_{21}\cos\phi \end{array}$$

where *σ*, *τ*, and *τ_L_* are the normal, longitudinal shear, and transverse shear stresses in the crack plane, respectively. *ϕ* denotes the angle of the fracture plane.

If the normal stress in the fracture plane is a tensile stress, i.e., *σ_N_*_;_ ≥ 0, then matrix tensile failure will occur; otherwise, matrix compression failure will occur. The matrix failure criteria are expressed as follows:

(18) $$f_{mat}=\left\{ \begin{array}{cc} f_{mt}={\left(\frac{\sigma_{N}}{Y_{T}}\right)}^{2}+{\left(\frac{\tau_{T}}{S_{T}}\right)}^{2}+{\left(\frac{\tau_{L}}{S_{L}}\right)}^{2}=1 & \sigma_{N}\geq 0\\ f_{mc}={\left(\frac{\tau_{T}}{S_{T}-\mu_{T}\sigma_{N}}\right)}^{2}+{\left(\frac{\tau_{L}}{S_{L}-\mu_{L}\sigma_{N}}\right)}^{2}=1 & \sigma_{N}<0 \end{array}\right.$$

where *Y_T_* is the transverse tensile strength; *μ_L_* and *μ_T_* are the friction coefficients in the longitudinal and transverse directions, respectively; and *S_L_* and *S_T_* are the in situ longitudinal and transverse shear strengths, respectively. It is worth noting that, although *S_L_* and *S_T_* are not the same as the parameters *S*_12_ and *S*_23_, respectively, used in physics, Puck et al. [31] found that the value of *S_L_* can be set to that of *S*_12_. The other parameters can be determined by analyzing the pure transverse compression of a composite laminate, which is calculated as follows [32]:

(19) $$\mu_{T}=-\frac{1}{\tan(2\phi_{0})},\;S_{T}=\frac{Y_{C}}{2\tan(\phi_{0})},\;\mu_{L}=\frac{\mu_{T}}{S_{T}}S_{L}$$

where *ϕ*_0_ is the angle of the fracture plane for pure compression [24,25], i.e., *ϕ*_0_ = 53 ± 2°, and *Y_C_* is the transverse compressive strength of the composite.

#### 2.3.3. Fiber Compression Failure

Schultheisz and Waas [26] observed a local matrix deformation accompanied by fiber fracture (i.e., “fiber kinking”) that differs from fiber microbuckling. Furthermore, Argon [27] assumed that initial microbuckling could result in fiber rotation, matrix shearing, fiber–matrix debonding, fiber kinking, and splitting in kink bands.

**Figure 2:**
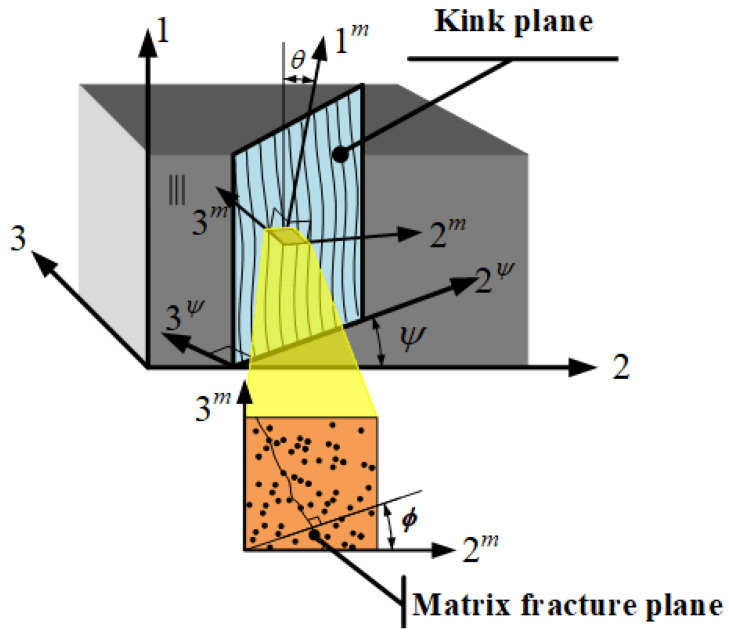
3D kinking model based on [32].

Although the fiber kinking mechanism is similar to that of matrix failure, a major difference is that in the matrix failure model, the associated stresses are calculated with respect to the fracture plane, whereas in fiber kinking analysis, stresses are calculated with respect to the kink plane, which is aligned with the fiber rotation. As shown in Figure 2, system 1–2–3 describes the material coordinates, where *ψ* is the angle between the kink plane and axis 2. Fiber kinking occurs on the plane with local coordinate system 1*^m^*–2*^m^*–3*^m^*, which is obtained by rotating the coordinate system 1–2*^ψ^*–3*^ψ^* by an angle *θ*.

The associated stress transformations in the transformation of the above coordinates are expressed as follows [28]:

(20) $$\left\{ \begin{array}{l} \sigma_{22}^{\psi }={\bar{\sigma }}_{22}\cos^{2}\psi +{\bar{\sigma }}_{33}\sin^{2}\psi +2{\bar{\sigma }}_{23}\sin\psi \cos\psi \\ \tau_{12}^{\psi }={\bar{\sigma }}_{12}\cos\psi +{\bar{\sigma }}_{31}\sin\psi \\ \tau_{23}^{\psi }=({\bar{\sigma }}_{33}-{\bar{\sigma }}_{22})\sin\psi \cos\psi +{\bar{\sigma }}_{23}\left(\cos^{2}\psi -\sin^{2}\psi \right)\\ \tau_{31}^{\psi }={\bar{\sigma }}_{31}\cos\psi -{\bar{\sigma }}_{12}\sin\psi \end{array}\right.$$

(21) $$\left\{ \begin{array}{l} \sigma_{22}^{m}={\bar{\sigma }}_{11}\sin^{2}\theta +\sigma_{22}^{\psi }\cos^{2}\theta -2\tau_{12}^{\psi }\sin\theta \cos\theta \\ \tau_{12}^{m}=(\sigma_{22}^{\psi }-{\bar{\sigma }}_{11})\sin\theta \cos\theta +\tau_{12}^{\psi }\left(\cos^{2}\theta -\sin^{2}\theta \right)\\ \tau_{23}^{m}=\tau_{23}^{\psi }\cos\theta -\tau_{31}^{\psi }\sin\theta \end{array}\right.$$

According to the concept of matrix compression failure, and taking the effect of matrix transverse tension due to fiber misalignment into account, the fiber kinking/splitting criterion can be expressed as [28]:

(22) $$f_{kink}=f_{split}={\left(\frac{\tau_{23}^{m}}{S_{T}-\mu_{T}\sigma_{22}^{m}}\right)}^{2}+{\left(\frac{\tau_{12}^{m}}{S_{L}-\mu_{L}\sigma_{22}^{m}}\right)}^{2}+{\left(\frac{\langle \sigma_{22}^{m}\rangle }{Y_{T}}\right)}^{2}$$

where $\sigma_{22}^{m}$, $\tau_{12}^{m}$, and $\tau_{23}^{m}$ are the transverse normal stress and the in-plane and out-of-plane shear stresses, respectively, in the coordinate system 1*^m^*–2*^m^*–3*^m^*, and <·> denotes the Macauley symbol, i.e., <x> = (x + |x|)/2. Pinho et al. [28] determined experimentally that fiber kinking takes place only for compressive stress ${\bar{\sigma }}_{11}$ ≤ −*X*_C_/2; otherwise, fiber splitting occurs.

Angle *ψ* is expressed by [32]:

(23) $$\tan(2\psi )=\frac{2{\bar{\sigma }}_{12}}{{\bar{\sigma }}_{22}-{\bar{\sigma }}_{33}}$$

The misalignment frame orientation *θ* is the sum of the initial misalignment angle *θ_i_* and the additional shear strain *γ* due to loading:

(24) $$\theta =\frac{\tau_{12}^{\psi }}{\left|\tau_{12}^{\psi }\right|}\left(\theta_{i}+\gamma \right)$$

The shear strain *γ* in the initial misalignment frame is defined as follows:

(25) $$\gamma =f_{CL}^{-1}\left(\left|-\frac{{\bar{\sigma }}_{11}-{\bar{\sigma }}_{22}}{2}\sin\left(2\theta_{i}\right)+\left|\tau_{12}^{\psi }\right|\cos\left(2\theta_{i}\right)\right|\right)$$

where *f_CL_* is the shear function (i.e., *τ* = *f_CL_*(*γ*)). For linear shear, Equation (25) can be simplified to:

(26) $$\gamma =\frac{\theta_{i}G_{12}+\left|{\bar{\sigma }}_{12}\right|}{G_{12}+{\bar{\sigma }}_{11}-{\bar{\sigma }}_{22}}-\theta_{i}$$

#### 2.3.4. Damage Propagation Criterion

**Figure 3:**
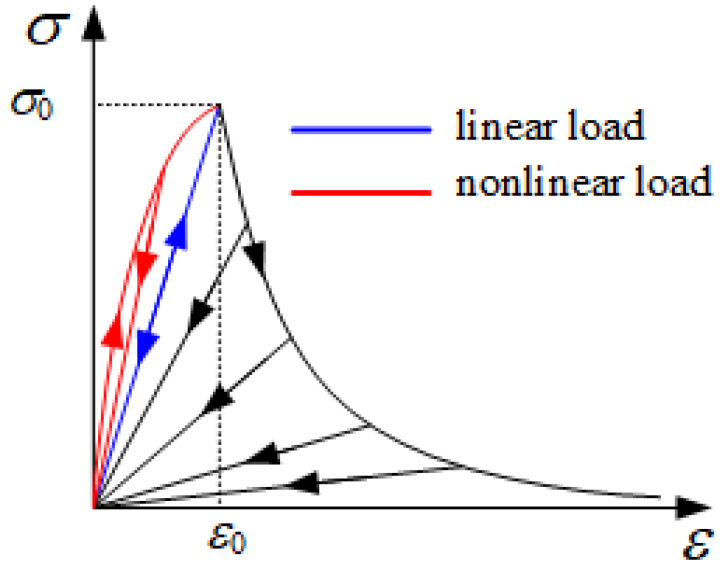
Loading/unloading and softening stress–strain curve [23].

Damage evolution is accompanied by release of strain energy and degradation of the material properties. The loading/unloading and softening stress–strain curves for a combined elastoplastic damage model are shown in Figure 3, where σ_0_ and ε_0_ are the initial values of the failure stress and strain in all directions, respectively.

Without considering plastic deformation in the fiber direction, the material exhibits a linear elastic behavior before damage. After damage occurs, the stiffness decreases gradually. Before damage is initiated, although irreversible plastic deformations in both the shear and transverse directions are observed, the material stiffness is not degraded. Therefore, a nonlinear behavior is shown during loading. Unlike in the fiber direction, both plastic deformation and stiffness softening occur beyond the point of damage initiation. Stiffness softening is expressed by an exponential damage parameter as follows [33]:

(27) $$d_{I}=1-\frac{1}{f_{I}}\exp\left[A_{I}\left(1-f_{I}\right)\right],\;I\in \left(mt,mc,ft,kink\;\mathrm{or}\;split\right)$$

A discrete element is the basic unit of the FE method. Although elements with different sizes obey the same stress–strain relationship, the energy release rates of different elements are unequal and proportional to the element size. To alleviate the dependence of the energy release rate on the element size, an element characteristic length *L_C_* is introduced with a critical strain energy release rate of *G*_I,C_. The damage energy dissipated per unit volume, *g*_I,C_, can be defined with the exponential factor *A_I_* as an internal variable [23] as follows:

(28) $$g_{I,C}\left(A_{I}\right)-\frac{G_{I,C}}{L_{C}}=0,I\in \left(mt,mc,ft,kink\;\mathrm{or}\;split\right)$$

where *G*_I,C_ contains *G_kink_*, *G_split_*, *G*_IC_, and *G*_IIC_, which are identical to the fiber kinking and splitting fracture toughness and modes I and II of the matrix fracture toughness, respectively. *g*_I,C_(*A_I_*) can be obtained from the following integration:

(29) $$g_{I,C}\left(A_{I}\right)=\int_{0}^{\infty }\frac{\partial \psi }{\partial d_{I}}\frac{d_{I}\left(A_{I}\right)}{dt}dt$$

By substituting Equation (29) into Equation (28), the following equation is derived, which is used to solve *A_I_* by numerical iterations:

(30) $$\ln\left(A_{I}^{\left(n+1\right)}\right)=\ln\left(A_{I}^{\left(n\right)}\right)-\ln\left(g_{I,C}\left(A_{I}^{\left(n\right)}\right)L_{C}/G_{I,C}\right)\frac{\ln\left(A_{I}^{\left(n\right)}/A_{I}^{\left(n-1\right)}\right)}{\ln\left(g_{I,C}^{\left(n\right)}/g_{I,C}^{\left(n-1\right)}\right)}$$

Details on the approach used to determine *A_I_* are provided in Chen et al. [23].

### 2.4. Cohesive Model

**Figure 4:**
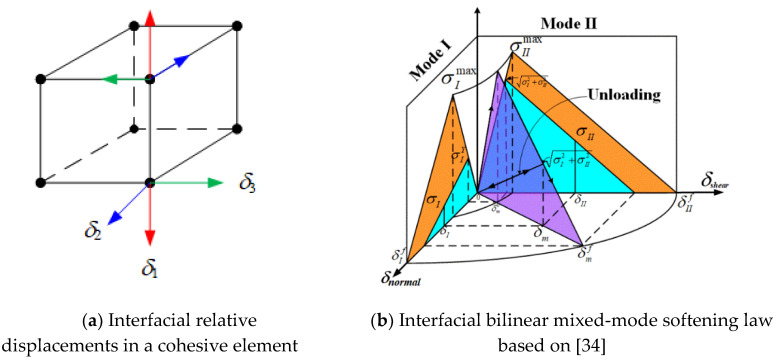
Cohesive zone.

A modified cohesive model [34] was adopted to simulate interfacial failure, as shown in Figure 4a. In this model, mode II and mode III fractures, which were considered as the combined resulting transverse shear modes, were used with mode I to compose the mixed mode, as shown in Figure 4b. The total mixed-mode relative displacement is expressed as:

(31) $$\delta_{m}=\sqrt{\delta_{I}^{2}+\delta_{II}^{2}}$$

where *δ*_Ι_ and *δ*_ΙΙ_ are defined as:

(32) $$\delta_{I}=\max(0,\delta_{1}),\;\delta_{II}=\sqrt{\delta_{2}^{2}+\delta_{3}^{2}}$$

Here, *δ*_1_ is the normal opening relative displacement, and *δ*_2,_ *δ*_3_ are the resulting transverse shear relative displacements.

A quadratic damage initiation criterion under a multiaxial stress state was used to predict the onset of delamination in the cohesive zone [34]:

(33) $$\sqrt{{\left(\frac{\max(\sigma_{I},0)}{\sigma_{I}^{\max}}\right)}^{2}+{\left(\frac{\sigma_{\mathrm{II}}}{\sigma_{\mathrm{II}}^{\max}}\right)}^{2}}=1$$

where $\sigma_{I}^{\max}$ and $\sigma_{\mathrm{II}}^{\max}$ denote the interlaminar tensile and shear strengths (see in Figure 4), respectively, and *σ*_Ι_ and *σ*_ΙΙ_ are the resulting normal interlaminar stress and shear stress of the interface, correspondingly. Assuming linear softening in the interface elements after the delamination onset, the fracture energy under mixed-mode loading with the power law criterion [35] is expressed as:

(34) $${\left(\frac{G_{I}}{G_{\mathrm{IC}}}\right)}^{\alpha }+{\left(\frac{G_{\mathrm{II}}}{G_{\mathrm{IIC}}}\right)}^{\alpha }=1$$

(35) $$d_{delam}=\max\left\{0,\min\left\{1,\frac{\delta_{m}-\delta_{m}^{0}}{\delta_{m}^{f}-\delta_{m}^{0}}\right\}\right\}$$

where *α* ∈[1.0, 2.0] is an empirical parameter derived from mixed-mode tests, and *G*_IC_ and *G*_IIC_ are the critical energy release rates for pure modes I (opening) and II (shear), respectively. The superscripts “0” and “*f*” indicate the initial and final values of the effective displacement *δ_m_*, respectively, and the max function represents the irreversible damage. 

## 3. Calculation of the Angle of the Fracture Plane

**Figure 5:**
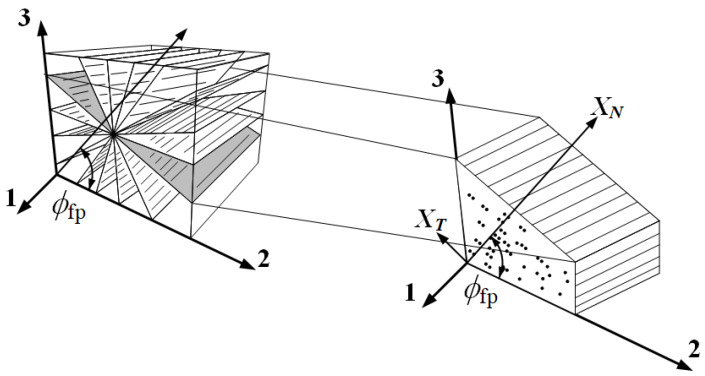
Search of fracture planes based on [31].

Knops [36] proposed an algorithm to determine the fracture plane in composite laminates. As shown in Figure 5, the fiber orientation of each ply in the laminate is assumed to be in direction 1. Subsequently, each ply is divided into discrete equal angular intervals of 1° from direction 1 (0°) to 180°. The stress components *σ_N_* (*ϕ*), *τ_T_*(*ϕ*), and *τ_L_*(*ϕ*) on the planes oriented at each angle are determined for a designated stress state and substituted into Equation (18) to derive the value of *f_mat_*. Then, the angle corresponding to the maximal *f_mat_* gives a potential orientation of the fracture plane. If *f_mat_* reaches 1.0 with increasing external loading, which indicates matrix failure initiation, then the associated angle of a potential fracture plane is the real orientation *ϕ*_fp_ of the fracture plane.

**Figure 6:**
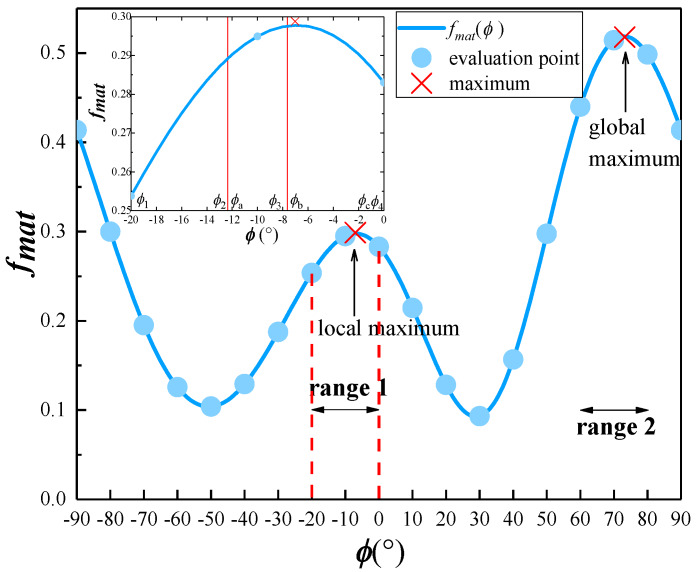
*f*_mat_ versus *ϕ* in the interval [−90°, 90°].

Although the procedure developed by Knops can be used to obtain a precise fracture angle, its efficiency is low. For a model with N elements and M increments, M × N × 180 iteration steps are required. Here, we propose a modified algorithm to determine the fracture angle with high efficiency and robustness based on the studies of Wiegand et al. [37] and Schirmaier et al. [38]. According to the research of Schirmaier et al. [38], the number of *f_mat_* thresholds does not exceed three, and the distance between two local maxima is always greater than 25°. Therefore, the fracture angle can be searched for in [−90°, 90°] with a step size of 10°, as shown in Figure 6. (If the curve increases or decreases monotonously, which is not shown in Figure 6, the fracture angle is 90° or −90°). The local extrema intervals (denoted as “range 1” and “range 2”) are determined by applying the golden section search method and an inverse interpolation.

For range 1 (see the magnified image at the top left corner of Figure 6), i.e., [*ϕ*_1_, *ϕ*_4_], the golden section points *ϕ*_2_ and *ϕ*_3_ can be determined as follows:

(36) $$\frac{\phi_{4}-\phi_{2}}{\phi_{2}-\phi_{1}}=\frac{\sqrt{5}+1}{2}$$

(37) $$\frac{\phi_{4}-\phi_{3}}{\phi_{3}-\phi_{2}}=\frac{\sqrt{5}+1}{2}$$

Subsequently, the values of *f_mat_* at *ϕ*_2_ and *ϕ*_3_ must be compared. If *f_mat_*(*ϕ*_2_) < *f_mat_*(*ϕ*_3_), the local extremum interval is substituted by [*ϕ*_2_, *ϕ*_4_]; otherwise, the local extremum interval is updated by [*ϕ*_1_, *ϕ*_3_]. For *f_mat_*(*ϕ*_2_) < *f_mat_*(*ϕ*_3_) in the current case, let *ϕ_a_* = *ϕ*_2_, *ϕ_c_* = *ϕ*_4_, and *ϕ_b_* = *ϕ*_3_. By applying an interpolation in interval [*ϕ_a_*, *ϕ_c_*], the function *f_mat_*(*ϕ*) is approximated as follows:

(38) $$\begin{array}{c} f_{mat}\left(\phi \right)\approx f_{mat}\left(\phi_{a}\right)\frac{\left(\phi -\phi_{b}\right)\left(\phi -\phi_{c}\right)}{\left(\phi_{a}-\phi_{b}\right)\left(\phi_{a}-\phi_{c}\right)}+f_{mat}\left(\phi_{b}\right)\frac{\left(\phi -\phi_{a}\right)\left(\phi -\phi_{c}\right)}{\left(\phi_{b}-\phi_{a}\right)\left(\phi_{b}-\phi_{c}\right)}\\ +f_{mat}\left(\phi_{c}\right)\frac{\left(\phi -\phi_{a}\right)\left(\phi -\phi_{b}\right)}{\left(\phi_{c}-\phi_{b}\right)\left(\phi_{c}-\phi_{a}\right)},\;\phi \in \left[\phi_{a},\phi_{c}\right] \end{array}$$

Therefore, the maximum point of range 1, *ϕ*_fp1_, is expressed as:

(39) $$\phi_{\mathrm{fp}1}\approx \phi_{b}-\frac{1}{2}\frac{{\left(\phi_{b}-\phi_{a}\right)}^{2}\left(f_{mat}\left(\phi_{b}\right)-f_{mat}\left(\phi_{c}\right)\right)-{\left(\phi_{b}-\phi_{c}\right)}^{2}\left(f_{mat}\left(\phi_{b}\right)-f_{mat}\left(\phi_{a}\right)\right)}{\left(\phi_{b}-\phi_{a}\right)\left(f_{mat}\left(\phi_{b}\right)-f_{mat}\left(\phi_{c}\right)\right)-\left(\phi_{b}-\phi_{c}\right)\left(f_{mat}\left(\phi_{b}\right)-f_{mat}\left(\phi_{a}\right)\right)}$$

The maximum point in range 2, denoted as *ϕ*_fp2_, is obtained by applying a similar procedure on range 2. Finally, the fracture angle in interval [−90°, 90°] is determined as the larger one of *ϕ*_fp1_ and *ϕ*_fp2_.

To verify the effectiveness of the above algorithm, four typical stress states were selected to determine the angle of the fracture plane: pure shear in-plane and out-of-plane, uniaxial compression, and arbitrary 3D stress, which are listed in Table 1. 

**Figure 7:**
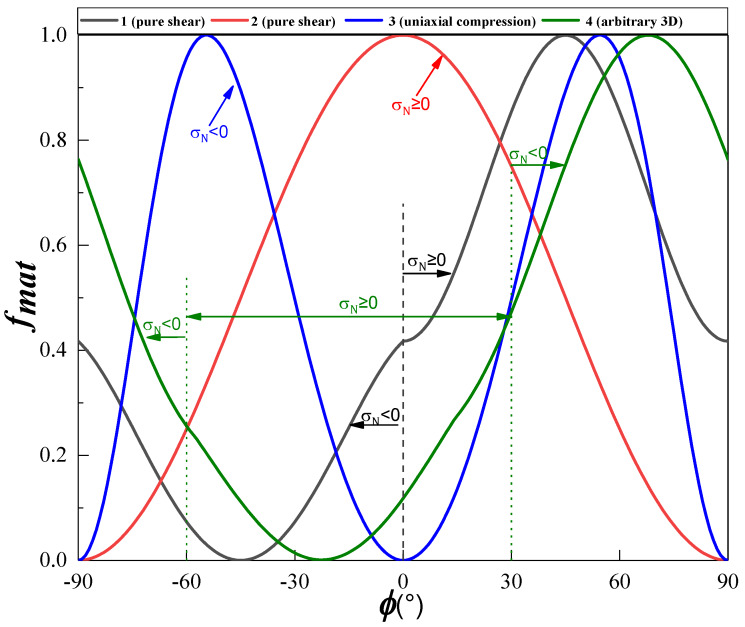
Threshold of matrix damage onset versus *ϕ* for four typical stress states.

The curves of the threshold of the matrix damage onset versus angle *ϕ* for IM7/8552 unidirectional composite laminates under the four typical stress states are shown in Figure 7. It can be seen that the fracture angles for the four cases are significantly different. For the case of uniaxial compression (i.e., curve 3), there are two peaks at *ϕ* = 54° and *ϕ* *=* −54°, while for the other three cases there is only one peak. For the two cases of pure shear, these peaks occur at *ϕ* = 0° and *ϕ* = 45° It is worth noting that an inflection point appears at *ϕ* = 0° in curve 1. In fact, for the case of pure shear associated with curve 1, the matrix tensile failure (i.e., σ_N_ ≥ 0) occurs in the interval [0°, 90°], while the matrix compressive failure (i.e., σ_N_ < 0) occurs in the interval [−90°, 0°]. Therefore, *ϕ* = 0° is the separation point for the tensile and compressive failure modes, which leads to an inflection point in the curve. Similar to curve 1, there is an inflection point in curve 4 at *ϕ* = −60°. This implies that for curve 4, the matrix failure is dominated by tensile stress in the interval [−60°, 30°] and by compressive stress in the intervals [−90°, −60°] and [30°, 90°].

A comparison between the proposed algorithm and Knops’ algorithm [36] in terms of efficiency and accuracy is shown in Table 2, where *N* is the number of calculation points and “time” represents the real calculation time. As shown in Table 2, the angles calculated using the proposed algorithm are similar to those derived with Knops’ algorithm with step size 0.1°, whereas the associated calculation time is reduced to between 1/10 and 1/15 of the latter. It can be concluded that the algorithm proposed here is highly accurate and efficient. 

## 4. VUMAT Subroutine

**Figure 8:**
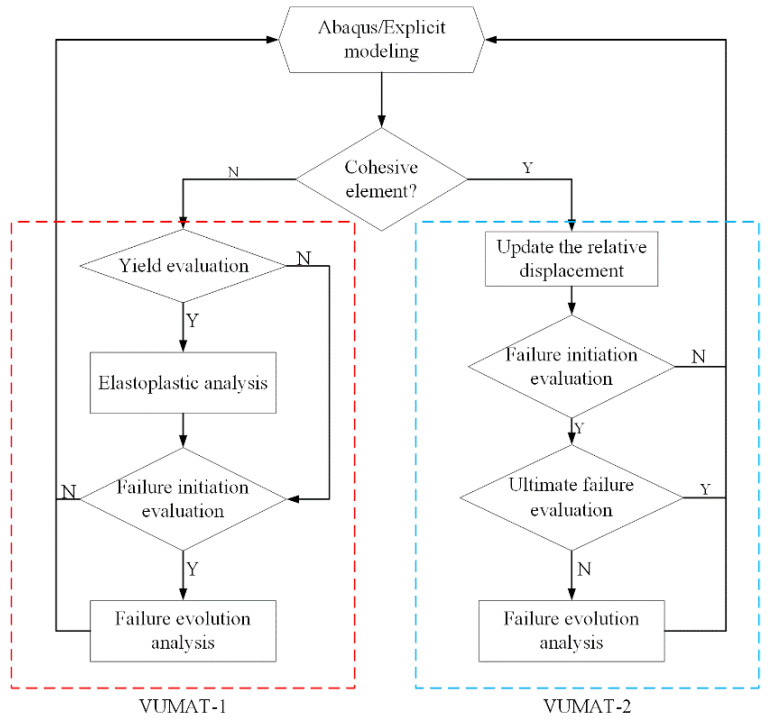
Flow chart of subroutine VUMAT.

Discontinuity problems do not easily converge in implicit solvers, such as damage problems and nonlinear problems. Hence, we developed a 3D elastoplastic damage algorithm implemented in the user-defined subroutine VUMAT as an extension to that of Chen et al. [23], which is suitable for a plane stress state. To accommodate compressive failure analysis, we replaced the LaRC criterion with the Hashin criterion [39]. In addition, the cohesive model was implemented in Abaqus/Explicit [40] using the user-defined subroutine VUMAT. The flow chart of the subroutine VUMAT is shown in Figure 8, which includes VUMAT-1 and VUMAT-2 for the elastoplastic damage and cohesive zone models, respectively. 

### 4.1. Elastoplastic Damage Algorithm

Similar to the study by Chen [23], the user-defined subroutine VUMAT of the elastoplastic damage algorithm was driven by a strain increment, in which the loading history was discretized into a sequence of time intervals, i.e., [*t_n_*, *t_n_*_+1_](*n* = 1, 2, 3). Therefore, the discrete backward Euler algorithm was applied to update the effective stress and strain components, and this process can be described as follows: the Abaqus/Explicit main program yields $\left\{\Delta \varepsilon_{n+1},\varepsilon_{n},\varepsilon_{n}^{e},\varepsilon_{n}^{p},{\tilde{\varepsilon }}_{n}^{p},\sigma_{n},{\bar{\sigma }}_{n},{\tilde{\sigma }}_{n},d_{1,n},d_{2,n},d_{3,n},\phi_{\mathrm{fp},n}\right\}$, a result of the *n* th increment, used as the initial condition to the *n*+1 th increment, which is updated to a novel set variable $\left\{\varepsilon_{n+1},\varepsilon_{n+1}^{e},\varepsilon_{n+1}^{p},{\tilde{\varepsilon }}_{n+1}^{p},\sigma_{n+1},{\bar{\sigma }}_{n+1},{\tilde{\sigma }}_{n+1},d_{1,n+1},d_{2,n+1},d_{3,n+1},\phi_{\mathrm{fp},n+1}\right\}$ at the end of the increment. Therefore, the incremental elastoplastic constitutive algorithm using the backward Euler explicit integration procedure is formulated as follows:

(40) $$\left\{ \begin{array}{l} \varepsilon_{n+1}=\varepsilon_{n}+\Delta \varepsilon_{n+1}\\ \varepsilon_{n+1}^{p}=\varepsilon_{n}^{p}+\Delta \lambda_{n+1}\partial_{{\bar{\sigma }}_{n+1}}F_{n+1}^{p}\\ \varepsilon_{n+1}^{e}=\varepsilon_{n+1}-\varepsilon_{n+1}^{p}\\ {\tilde{\varepsilon }}_{n+1}^{p}={\tilde{\varepsilon }}_{n}^{p}+\Delta \lambda_{n+1}\\ {\bar{\sigma }}_{n+1}=C_{0}:\varepsilon_{n+1}^{e}\\ F_{n+1}=F\left({\bar{\sigma }}_{n+1},{\tilde{\varepsilon }}_{n+1}^{p}\right)\leq 0 \end{array}\right.$$

The nonlinearity in Equation (40) can be solved by applying the Newton–Raphson method. The iteration was performed until the yield criterion $F\left({\bar{\sigma }}_{n+1}^{k+1},{\tilde{\varepsilon }}_{n+1}^{p,\left(k+1\right)}\right)\leq tol$ was satisfied at the *k*+1 th iteration, where *tol* is the error tolerance of 1 × 10^−6^. The implementation procedure is as follows:

(1) Initial conditions:
  $$\Delta \varepsilon_{n+1},\varepsilon_{n},\varepsilon_{n}^{e},\varepsilon_{n}^{p},{\tilde{\varepsilon }}_{n}^{p},\sigma_{n},{\bar{\sigma }}_{n},{\tilde{\sigma }}_{n},d_{1,n},d_{2,n},d_{3,n},\phi_{\mathrm{fp},n.}$$
(2) Elastic predictor:
  (41) $$\left\{ \begin{array}{l} \varepsilon_{n+1}^{}=\varepsilon_{n}+\Delta \varepsilon_{n+1}\\ \varepsilon_{n+1}^{p,trial}=\varepsilon_{n}^{p}\\ \varepsilon_{n+1}^{e,trial}=\varepsilon_{n+1}^{}-\varepsilon_{n+1}^{p,trial}=\varepsilon_{n}^{e}+\Delta \varepsilon_{n+1}\\ {\tilde{\varepsilon }}_{n+1}^{p,trial}={\tilde{\varepsilon }}_{n}^{p}\\ {\tilde{\sigma }}_{n+1}^{trial}={\tilde{\sigma }}_{n}\\ {\bar{\sigma }}_{n+1}^{trial}={\bar{\sigma }}_{n}+C_{0}:\Delta \varepsilon_{n+1} \end{array}\right.$$
(3) Yield judgment and plastic corrector:
  (42) $$F_{n+1}^{trial}=F^{p}\left({\bar{\sigma }}_{n+1}^{trial}\right)-\tilde{\sigma }\left({\tilde{\varepsilon }}_{n+1}^{p,trial}\right)\leq 0$$
  If $F_{n+1}^{trial}$, then
  (43) $$\begin{array}{l} \varepsilon_{n+1}^{e}=\varepsilon_{n+1}^{e,trial},\varepsilon_{n+1}^{p}=\varepsilon_{n}^{p},{\tilde{\varepsilon }}_{n+1}^{p}={\tilde{\varepsilon }}_{n}^{p}\\ {\bar{\sigma }}_{n+1}={\bar{\sigma }}_{n+1}^{trial},{\tilde{\sigma }}_{n+1}={\tilde{\sigma }}_{n} \end{array}$$
  Else
  a. initialize parameter:
    (44) $$k=0,\Delta \lambda_{n+1}^{\left(0\right)}=0,{\bar{\sigma }}_{n+1}^{\left(0\right)}={\bar{\sigma }}_{n+1}^{trial},{\tilde{\varepsilon }}_{n+1}^{p,\left(0\right)}={\tilde{\varepsilon }}_{n+1}^{p,trial},\varepsilon_{n+1}^{p,\left(0\right)}=\varepsilon_{n+1}^{p,trial}$$
  b. calculate $F_{n+1}^{\left(k+1\right)}=F\left(\Delta \lambda_{n+1}^{\left(k+1\right)}\right)=F\left(\bar{\sigma }\left(\Delta \lambda_{n+1}^{\left(k+1\right)}\right),{\tilde{\varepsilon }}^{p}\left(\Delta \lambda_{n+1}^{\left(k+1\right)}\right)\right)$,
  c. do while $F_{n+1}^{\left(k+1\right)}\geq tol$ then
    (45) $$\begin{array}{l} \delta \left(\Delta \lambda_{n+1}^{\left(k+1\right)}\right)=-\frac{F\left(\Delta \lambda_{n+1}^{\left(k\right)}\right)}{F^{\prime }\left(\Delta \lambda_{n+1}^{\left(k\right)}\right)}\\ \Delta \lambda_{n+1}^{\left(k+1\right)}=\Delta \lambda_{n+1}^{\left(k\right)}+\delta \left(\Delta \lambda_{n+1}^{\left(k+1\right)}\right)\\ {\tilde{\varepsilon }}_{n+1}^{p,\left(k+1\right)}={\tilde{\varepsilon }}_{n+1}^{p,\left(k\right)}+\delta \left({\tilde{\varepsilon }}_{n+1}^{p}\right)\\ {\bar{\sigma }}_{n+1}^{\left(k+1\right)}=C_{0}:\left(\varepsilon_{n+1}-\varepsilon_{n+1}^{p,\left(k+1\right)}\right)\\ F_{n+1}^{\left(k+1\right)}=F\left({\bar{\sigma }}_{n+1}^{\left(k+1\right)},{\tilde{\varepsilon }}_{n+1}^{p,\left(k+1\right)}\right)=F\left(\bar{\sigma }\left(\Delta \lambda_{n+1}^{\left(k+1\right)}\right),{\tilde{\varepsilon }}^{p}\left(\Delta \lambda_{n+1}^{\left(k+1\right)}\right)\right)\\ k=k+1 \end{array}$$
    End do
    End.
(4) Damage judgment and corrector:
  a. Search for the fracture plane:
    If $f_{mat,n}<1$ then
    Calculate $\phi_{\mathrm{fp},n+1}$ (Equation (39))
    Else
    (46) $$\phi_{\mathrm{fp},n+1}=\phi_{\mathrm{fp},n}$$
    End.
  b. Calculate the effective stress on the fracture plane $\sigma_{N}$, $\tau_{L}$, and $\tau_{T}$ (Equation (17)) and the effective stress in the fiber misalignment frame $\sigma_{22}^{m}$, $\tau_{12}^{m}$, and $\tau_{23}^{m}$ (Equation (21)).
  c. Damage judgment:
    (1) Check the fiber failure criterion:
      If ${\bar{\sigma }}_{11,n+1}\geq 0$ then
      Calculate $f_{ft}$ (Equation (16)) and $d_{ft}$
      Else
      Calculate $f_{kink}$ or $f_{split}$ Equation (22) and $d_{fc}$
      End.
    (2) Check the matrix failure criterion:
      If $\sigma_{N,n+1}\geq 0$ then
      Calculate $f_{mt}$ (Equation (18)) and $d_{mt}$
      Else
      Calculate $f_{mc}$ (Equation (18)) and $d_{mc}$
      End.
(5) Correct the nominal Cauchy stress:
  a. Update the state dependent variables:
    (47) $$\begin{array}{l} d_{1,n+1}=\max(d_{1,n},d_{ft}\mathrm{or}d_{fc})\\ d_{2,n+1}=\max(d_{2,n},d_{mt}\mathrm{or}d_{mc})\\ d_{3,n+1}=1-\left(1-d_{1,n+1}\right)\left(1-d_{2,n+1}\right) \end{array}$$
  b. Calculate the nominal stress tensor $\sigma_{n+1}$ (Equation (6)).

### 4.2. Cohesive Zone Algorithm

The numerical algorithm for the cohesive zone implemented in the user-defined subroutine VUMAT is based on [34]. According to the flow chart of VUMAT-2 in Figure 8, the algorithm primarily comprises the calculation of the relative displacement, evaluation of initial and ultimate failure, and derivation of the damage parameter *d*, which reflects the failure evolution. Therefore, the details of FE implementation in the cohesive zone are as follows:

(1) Obtain material properties: $K_{I},K_{II},\sigma_{I}^{\max},\sigma_{II}^{\max},G_{IC},G_{IIC},T_{0}$.
(2) Relative displacement computation:
  - Calculate the relative displacement in the local orthogonal coordinate system:
    (48) $$\delta_{i,n+1}=T_{0}\left(\varepsilon_{i,n}+\Delta \varepsilon_{i,n+1}\right)\;i=1,2,3$$
  - Calculate the mixed-mode relative displacement $\delta_{m}$ and its components:
    (49) $$\delta_{I,n+1}=\max\left(0,\delta_{1,n+1}\right),\;\delta_{II,n+1}=\sqrt{\delta_{2,n+1}^{2}+\delta_{3,n+1}^{2}},\;\delta_{m,n+1}=\sqrt{\delta_{I,n+1}^{2}+\delta_{II,n+1}^{2}}$$
  - Calculate the relative displacement of mixed mode $\delta_{m,n+1}^{0}$ at the initial failure using Equation (33):
    (50) $$\delta_{m,n+1}^{0}=1/\sqrt{{\left(\frac{\langle K_{I}\delta_{I,n+1}/\delta_{m,n+1}\rangle }{\sigma_{I}^{\max}}\right)}^{2}+{\left(\frac{K_{II}\delta_{II,n+1}}{\delta_{m,n+1}\sigma_{II}^{\max}}\right)}^{2}}$$
(3) Verify the initial failure criterion:
  (51) $$f_{initial}=\delta_{m,n+1}-\delta_{m,n+1}^{0}\leq 0$$
  If $f_{initial}\leq 0$, then set *d_n_*_+1_ = 0; otherwise, the damage evolves. Therefore, calculate the relative displacement of mixed mode $\delta_{m,n+1}^{f}$ at the ultimate failure using Equation (34):
  (52) $$\delta_{m,n+1}^{f}={\left({\left(\frac{K_{I}\delta_{m,n+1}^{0}{\left(\delta_{I}\right)}^{2}}{2G_{IC}{\left(\delta_{m}\right)}^{2}}\right)}^{\alpha }+{\left(\frac{K_{II}\delta_{m,n+1}^{0}{\left(\delta_{II}\right)}^{2}}{2G_{IC}{\left(\delta_{m}\right)}^{2}}\right)}^{\alpha }\right)}^{-1/\alpha }$$
(4) Verify the ultimate failure criterion:
  (53) $$f_{ultimate}=\delta_{m,n+1}-\delta_{m,n+1}^{f}\leq 0$$
  If $f_{ultimate}\leq 0$, then set $d_{n+1}=\frac{\delta_{m,n+1}-\delta_{m,n+1}^{0}}{\delta_{m,n+1}^{f}-\delta_{m,n+1}^{0}}$. Otherwise, $d_{n+1}=$ 1.
(5) Update the nominal Cauchy stress:
  (54) $$\sigma_{1,n+1}=\left(1-d\right)K_{I}\delta_{1,n+1},\;\sigma_{2,n+1}=\left(1-d\right)K_{II}\delta_{2,n+1},\;\sigma_{3,n+1}=\left(1-d\right)K_{II}\delta_{3,n+1}$$

## 5. Numerical Examples

To verify the elastoplastic damage model developed in this study, numerical simulations of the progressive failure of composite laminates with fiber waviness were performed. Mukhopadhyay et al. [10] conducted an experimental and numerical study of the compressive failure of IM7/8552 laminates with wrinkles. For comparison, the IM7/8552 laminates with a layup of [45_2_/90_2_/−45_2_/0_2_]_3s_ used by Mukhopadhyay et al. [10] were selected in the present simulation. Thus, the numerical and experimental results in [10,41] can be used for comparison with the results obtained from the proposed elastoplastic damage model. The geometry of the laminates and the profile of the embedded wrinkles are described in the following.

The elastic properties [10] and plastic parameters [22] are listed in Table 3, where the plastic parameters *a*_66_, *β* and *m* were converted using Equation (11).

**Figure 9:**
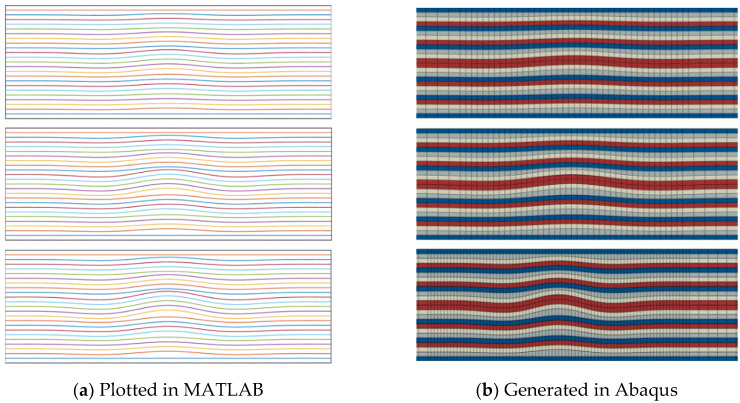
Section of wrinkle level 5.6°, 9.9°, and 11.4° from top to bottom.

First, the profile of the embedded wrinkles was plotted in MATLAB; subsequently, the associated FE model was generated using a Python script run by Abaqus GUI, shown in Figure 9. The profile of the wrinkles can be described with a cosine function as follows [10]:

(55) $$\left\{ \begin{array}{l} h_{w}=h_{0}+\Delta h\\ \Delta h=\left\{ \begin{array}{ll} \frac{B\delta }{2}\cos\left(\frac{2\pi x}{L}\right) & \;\mathrm{for}\;-\frac{L}{2}\leq x\leq \frac{L}{2}\\ 0 & \;\mathrm{otherwise} \end{array}\right. \end{array}\right.$$

where *h_w_* is the nodal coordinate of the through-thickness in the wrinkled configuration; *h*_0_ is the nodal coordinate in a wrinkle-free flat laminate; *L* is the wavelength of the wrinkle; and *δ* is the wave amplitude. The value of *B* is unity on the centerline and decreases linearly with the thickness, with a ratio of 1.0:0.63:0.39:0.0 [42].

**Figure 10:**
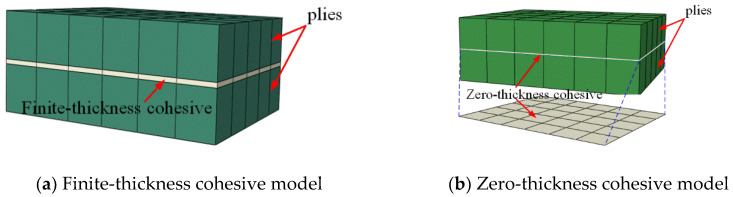
Cohesive models.

A 3D quasi-static progressive failure simulation was implemented in Abaqus/Explicit using the user-defined subroutine VUMAT. To reduce the computational cost, two adjacent plies with the same orientation were modeled using continuum 3D eight-noded reduced integration (C3D8R) elements, with one element through the thickness of the laminate, without considering delamination failure between those two plies. Layers of eight-noded 3D cohesive (COH3D8) interface elements with zero thickness were inserted between the plies at different orientations to model delamination failure, with the cohesive material properties shown in Table 4. Usually, there are cohesive elements with zero and finite thickness, as shown in Figure 10. The roles of the two types of cohesive element are identical. However, a finite-thickness cohesive element must be meshed using extremely small thickness intervals, which greatly increases the computation time in Abaqus/Explicit. To avoid extremely dense grids in thickness, which reduce the computing efficiency, zero-thickness elements were used in the present work.

**Figure 11:**
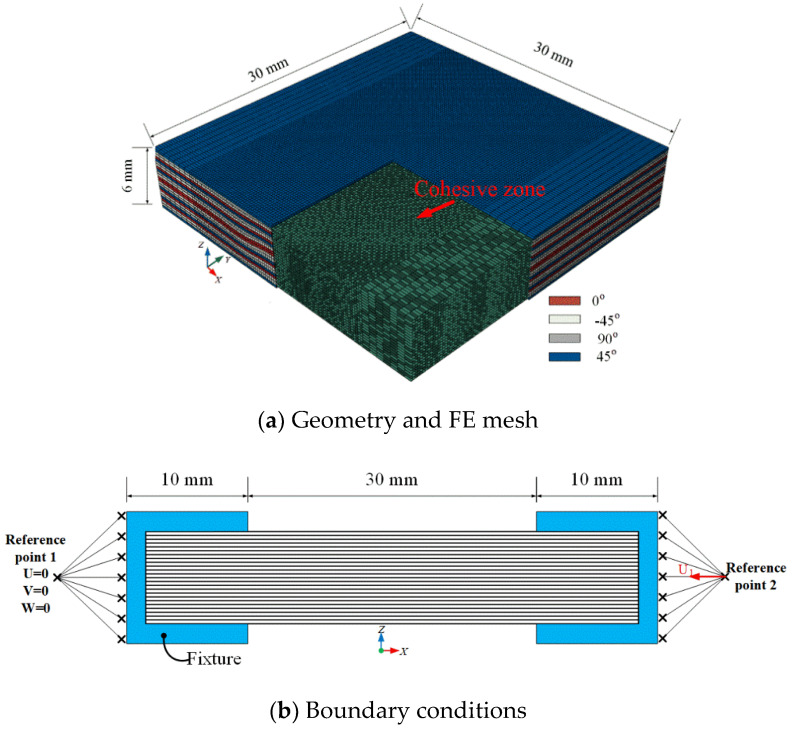
Geometry and boundary conditions of the wrinkle laminate.

The laminate dimensions were 30 × 30 × 6 mm, with a nominal ply thickness of 0.125 mm, as shown in Figure 11a. A fine mesh with in-plane dimensions of 0.25 × 0.25 mm was applied at the location of the wrinkle and towards the laminate edges, whereas comparatively coarser meshes of dimensions 0.5 × 0.25 mm and 0.75 × 0.25 mm were used elsewhere for computational efficiency. A typical model of the compression specimen comprised approximately 270,000 C3D8R elements and 260,000 COH3D8 elements. The boundary conditions shown in Figure 11b are as follows: fully constrained boundary conditions were applied on the left end (fixed) through reference point 1, and displacement boundary conditions were applied to the load direction by constraining the other directions to reference point 2 on the right end.

### 5.1. Compressive Failure Stress

**Figure 12:**
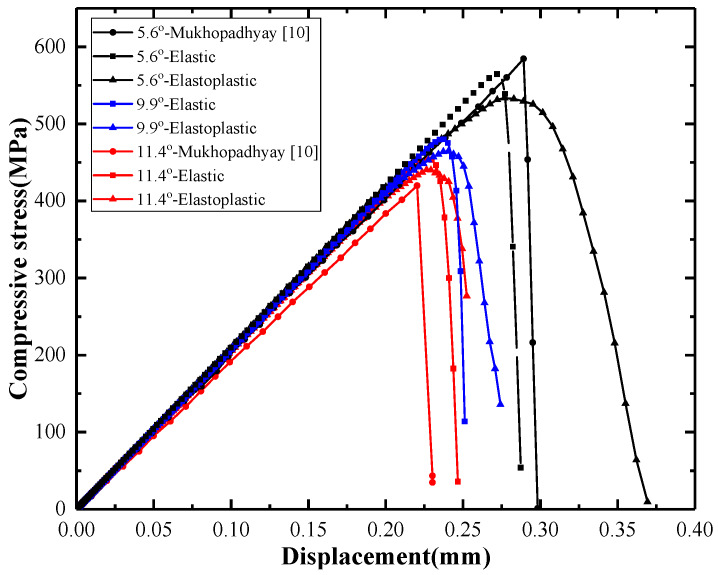
Predicted compressive stress versus displacement, obtained using three different models

Three levels of wrinkle severity, with a maximum waviness angle of 5.6°, 9.9°, and 11.4°, were investigated. The predicted compressive stresses versus the displacement curves calculated using three models are illustrated in Figure 12. As shown, both the predicted stiffness and the strength decrease with increasing wrinkle level for the three models. The stiffness predicted by the elastic model is the highest, while that obtained with the model described in [10] is the lowest. The strength calculated with the model of [10] is higher than that derived with the elastic model and the proposed elastoplastic model for defect angle 5.6°, but an opposite trend is observed for the higher angle of 11.4°. The model in [10] considers the effects of both shear nonlinearity and residual thermal stress. The above opposite trends might be an effect of the residual thermal stress on the stiffness and strength of laminates with different wrinkle levels. It can be seen that the displacement at final failure predicted by the elastoplastic model is significantly greater than those of the other two models, owing to the plastic effects.

**Figure 13:**
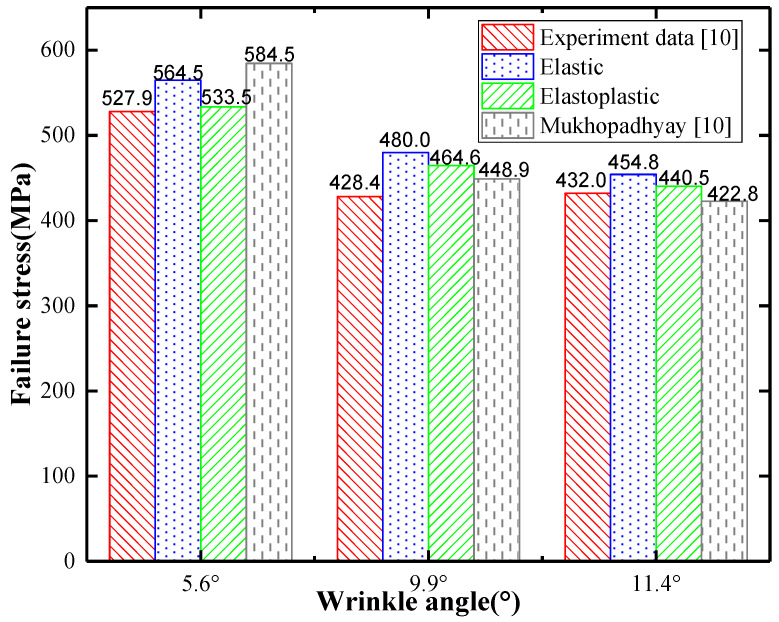
Compressive strength comparison for the three models and test results.

To demonstrate the effectiveness and predictive accuracy of the elastoplastic model, the compressive strengths predicted by the three models for different wrinkle severities are compared with test data [10,41] in Figure 13. As shown, the discrepancy between the results of the elastoplastic model and the test data for the three levels of wrinkle severity are 1.07%, 8.45%, and 1.97%. The errors of the elastic model are 6.93%, 12.04%, and 5.28%, and the prediction errors of the model in [10] are 10.73%, 3.64%, and −2.79%. The elastoplastic model provided better predictions than the elastic model. Compared with the model proposed in [10], the elastoplastic model achieved more accurate results for wrinkle levels 5.6° and 11.4°. Theoretically, the compressive strength of the laminate with wrinkle level 9.9° should be greater than that of wrinkle level 11.4°, but the opposite was observed in the experiment reported in [10] (as shown in Figure 13). The authors of [10] explained in [41] that this test result was a statistical average value of the compressive strength. They found that the test error for wrinkle level 9.9° was greater than those for 5.6° and 11.4°, owing to the strong fluctuations in the dataset obtained at wrinkle level 9.9°. This may be the reason that the model in [10] seems superior to the elastoplastic model in the compressive strength comparison for wrinkle level 9.9°.

**Figure 14:**
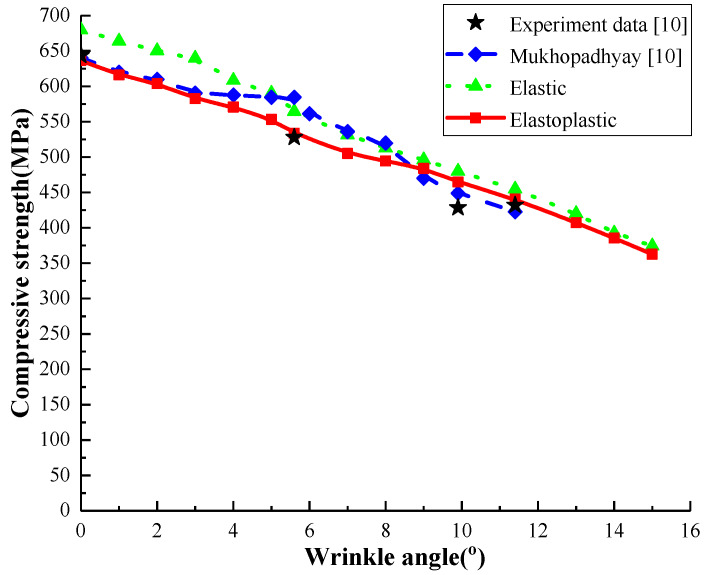
Wrinkle severity versus compressive strength.

The predicted curves of compressive strength versus the wrinkle severity by the three models were shown in Figure 14. As can be seen, the computed compressive strengths by the elastoplastic model match better with the measured data [10] than those by the other two models. It should be noted that the discrepancy between the prediction by the elastoplastic model and the elastic model decreases with the increase of wrinkle severity.

### 5.2. Failure Mechanism

To further demonstrate the advantages of considering the elastoplastic effect, the damage evolution is examined in this section. Mukhopadhyay et al. [10] used high-speed imaging to record the failure behavior of IM7/8552 composite laminates with fiber waviness subjected to compressive loading.

**Figure 15:**
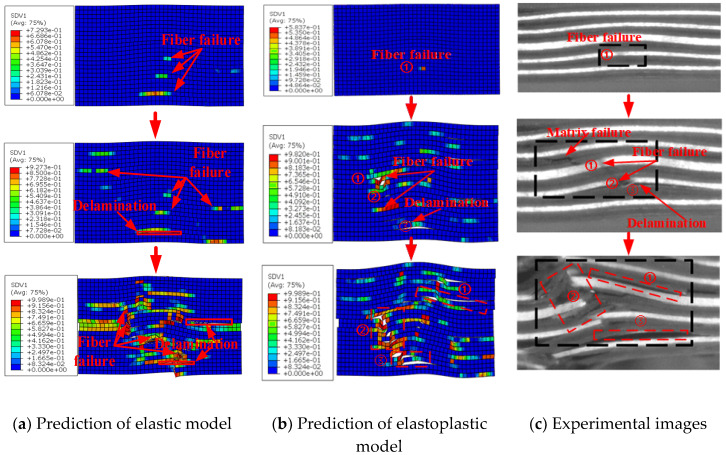
Damage evolution in a specimen with wrinkle level 5.6°. (**a**) Predicted by the elastic model, (**b**) predicted by the elastoplastic model, and (**c**) from high-speed camera images of the damage sequence in [10].

Figure 15a,b show the damage evolution at wrinkle level 5.6° predicted by the elastic model and the elastoplastic model, respectively, and the corresponding high-speed camera images of the damage sequence reported in [10] are shown in Figure 15c. It can be seen that the predictions of the elastoplastic model agree better with the test images than those of the elastic model. According to the prediction of the elastoplastic model and the test images, fiber failure first occurred in one of the 0° plies, followed by delamination in the interface between the 90° and −45° plies. Subsequently, further fiber failure and through-thickness delamination occurred when the loading was increased. It is clear that the damage locations predicted by the proposed model match well with those in the test images.

**Figure 16:**
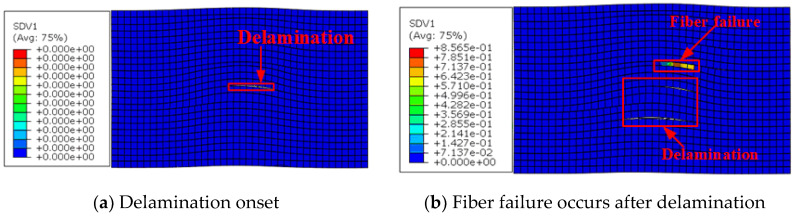
Elastoplastic model prediction of damage sequence for a laminate with wrinkle level 9.9°.

**Figure 17:**
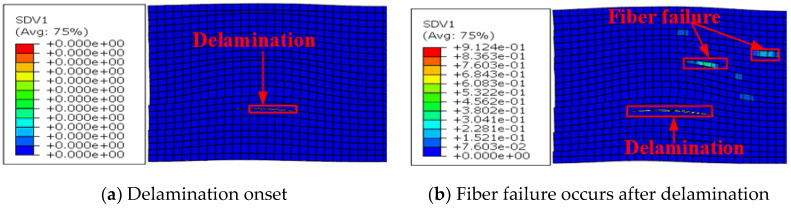
Elastoplastic model prediction of damage sequence for a laminate with wrinkle level 11.4°.

The elastoplastic damage prediction results for laminates of wrinkle levels 11.4° and 9.9° are shown in Figure 16 and Figure 17, respectively. The same damage sequence was observed in these two cases. Inter-ply delamination initiated earlier than fiber failure for the wrinkle severity levels 11.4° and 9.9°, in contrast to the case of wrinkle level 5.6°. This is in accordance with the results of Mukhopadhyay et al. [10]; they reported that fiber compressive failure was the dominant mechanism for lower-severity wrinkles (wrinkle severity lower than 8.7° for the laminates discussed), whereas inter-ply delamination was the driving mode of damage for higher-severity wrinkles (wrinkle severity exceeding 8.7° for the laminates discussed). 

**Figure 18:**
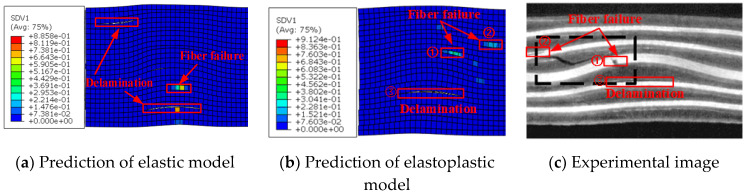
Damage locations for a specimen with wrinkle level 11.4° (**a**) predicted by the elastic model, (**b**) predicted by the proposed elastoplastic model, and (**c**) test image [10].

Figure 18 shows the damage location prediction of a laminate with wrinkle level 11.4°. As shown, similar to the case of wrinkle level 5.6°, the fiber failure and delamination locations obtained with the elastoplastic model match better with those in the test image than the elastic model. 

### 5.3. Plastic Effects

**Figure 19:**
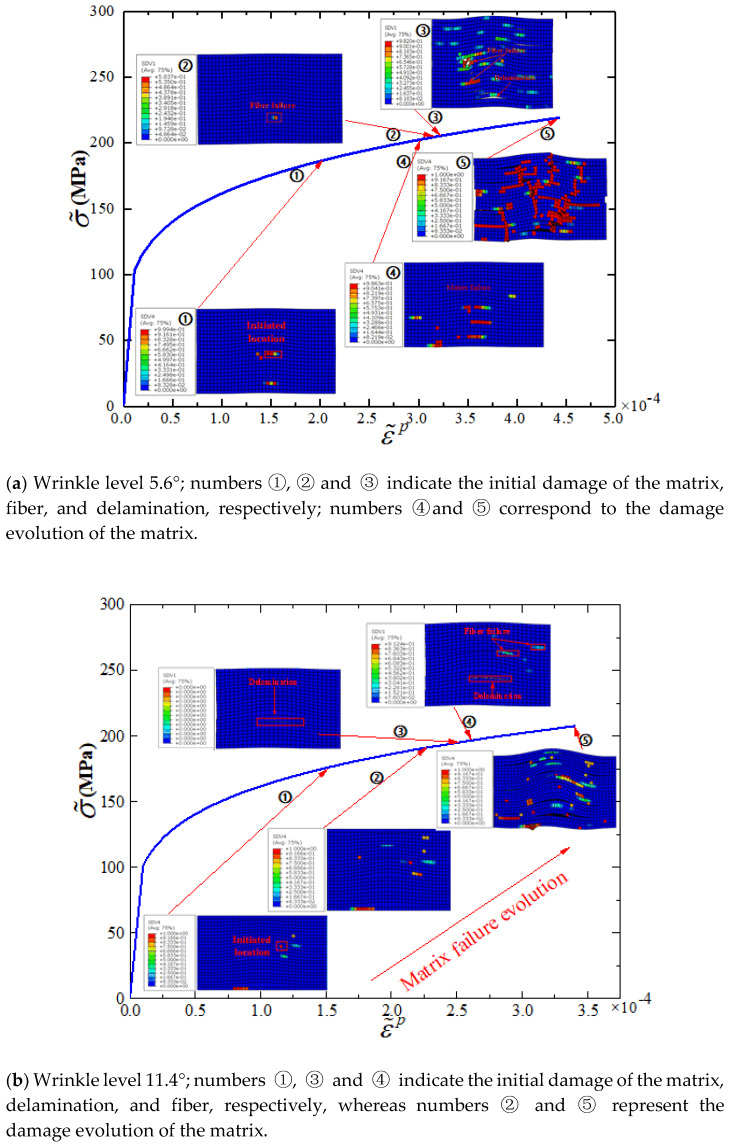
Equivalent stress versus equivalent plastic strain for wrinkle levels 5.6° and 11.4°.

The curves of equivalent stress versus equivalent strain for the location of the matrix damage initiation are shown in Figure 19, with clusters of the damage evolution sequence shown together. As shown in Figure 19a,b, matrix damage was initiated in phase ①, where the associated equivalent plastic strains were 2.032 × 10^−4^ and 1.5 × 10^−4^. This indicates that plasticity occurred before damage initiation. The matrix damage was initiated at the location of the maximum misalignment angle for these two cases, as shown in the damage cloud in phase ①. As loading proceeded, regions of fiber wrinkles underwent deformation owing to the large strain in the matrix and then triggered the next damage mode, i.e., fiber failure (see Figure 19a in phase ②) or delamination (see Figure 19b in phase ③). At the later stage of damage development, the strains experienced within the kind band were large (4.43 × 10^−4^ and 3.42 × 10^−4^ for wrinkle levels 5.6° and 11.4°, respectively), as shown in phase ⑤. This is similar to the compressive failure mechanism in unidirectional composite laminates [11]. The nonlinear deformation characteristics of the matrix were described well by the proposed elastoplastic damage model, which could not be achieved with the elastic damage model.

**Figure 20:**
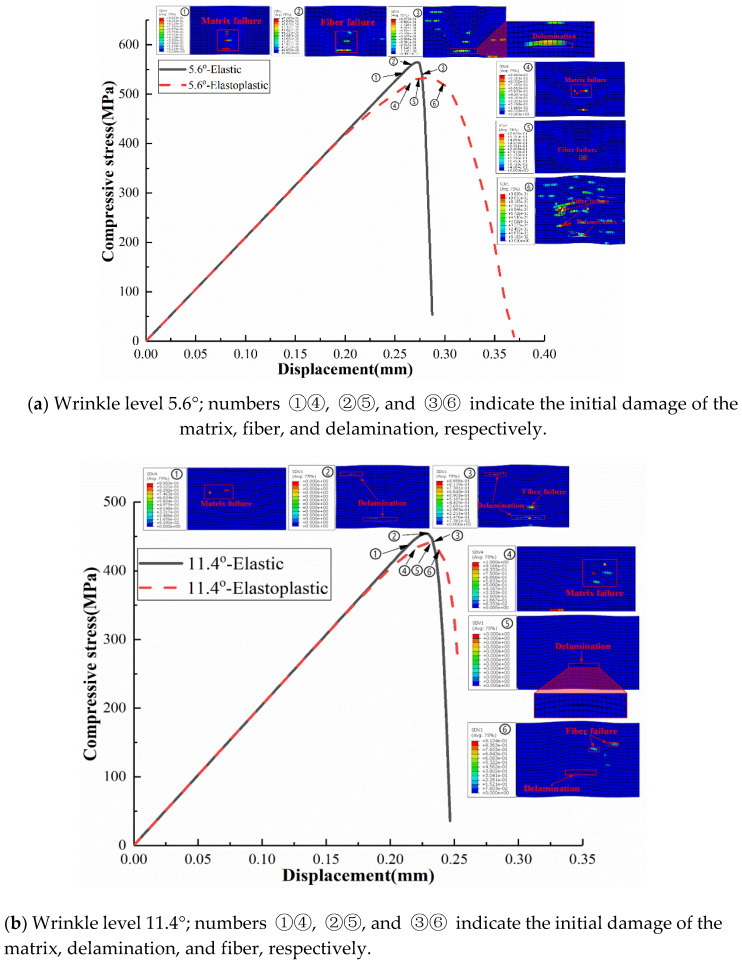
Predicted compressive stress versus displacement for wrinkle levels 5.6° and 11.4°. Numbers ①②③ indicate the damage initiation obtained from the elastic model, whereas ④⑤⑥ indicate that derived from the elastoplastic model.

For comparison, the predicted compressive stress versus displacement curves obtained using the elastic model and the elastoplastic model are plotted in Figure 20 for wrinkle levels 5.6° and 11.4°, with clouds of damage development sequences shown together. For the two wrinkle levels, the initiations of matrix damage, fiber failure, and delamination were delayed in the elastoplastic model compared with the elastic model. It is worth noting that the ultimate displacement at final failure for wrinkle level 5.6° was larger than that for 11.4°. This indicates that the laminate with wrinkle level 5.6° is relatively ductile, whereas that with wrinkle level 11.4° exhibits a brittle behavior. Moreover, the discrepancy between the ultimate displacements obtained from the elastic model and the elastoplastic model for wrinkle level 5.6° are much greater than those for wrinkle level 11.4°. Combined with the predicted curves of compressive strength versus wrinkle severity in Figure 14, it may imply that the plastic effect should not be neglected for laminates with lower wrinkle levels, at least for the laminate with the ply sequence discussed herein.

## 6. Conclusions

The progressive failure of multidirectional fiber-reinforced polymer laminates with embedded wrinkle defects was numerically simulated using an elastoplastic damage model. Damage evolution analysis was performed according to the LaRC05 criterion with four damage modes (i.e., fiber tensile failure, matrix failure, fiber kinking/splitting, and delamination). A modified algorithm with high efficiency and robustness was proposed based on a previous study to rapidly search for the fracture angle in the matrix failure analysis. This algorithm achieves high accuracy and significantly improved computational stability by combining the golden section search method and an inverse interpolation. The elastoplastic damage model was applied to simulate the compressive failure behavior of IM7/8552 [45_2_/90_2_/−45_2_/0_2_]_3s_ composite laminates with out-of-plane wrinkles. The results showed that the model can reproduce the nonlinearity of the laminate during the evolution of the damage and provide more accurate compressive strength predictions than the elastic model and a previous model [10]. In addition, the proposed model could determine the damage locations during the progressive failure process by comparing with test images. Based on the comparison of the compressive stress–displacement curves predicted by the elastoplastic and elastic models, it can be concluded that plasticity effects should not be neglected for laminates with low wrinkle levels.

## Figures and Tables

**Table 1:** Four typical stress states for IM7/8552 composite (units: MPa).

Stress State	*σ*_11_	*σ*_22_	*σ*_33_	*τ*_12_	*τ*_23_	*τ*_13_
1 (pure shear)	0.0	0.0	0.0	0.0	73	0.0
2 (pure shear)	0.0	0.0	0.0	94.195	0.0	0.0
3 (uniaxial compression)	0.0	−316.8	0.0	0.0	0.0	0.0
4 (arbitrary 3D)	0.0	−20	50	21	43.3	32.57

**Table 2:** Comparison of the efficiency and accuracy of the proposed algorithm with those of Knops’ algorithm.

Stress State	Knops’s Algorithm				Proposed Algorithm		
	Step Size (^o^)	*ϕ*_fp_ (^o^)	*N*	Time (s)	*ϕ*_fp_ (^o^)	*N*	Time (s)
**1 (pure shear)**	1	45	180	0.0625	45.0114	22	0.0113
	0.1	45	1800	0.1250			
**2 (pure shear)**	1	0	180	0.0781	4.44 × 10^−16^	22	0.0142
	0.1	0	1800	0.2344			
**3 (uniaxial compression)**	1	54	180	0.0625	54.271	25	0.0156
	0.1	54.5	1800	0.2561			
**4 (arbitrary 3D)**	1	68	180	0.0732	67.8691	22	0.0156
	0.1	67.9	1800	0.1094			

**Table 3:** Material properties of IM7/855 and model parameters [10,22].

***E*** **_11_ (MPa)**	***E*** **_22_ (MPa)**	***E*** **_33_ (MPa)**	***G*** **_12_ (MPa)**	***G*** **_13_ (MPa)**	***G*** **_23_ (MPa)**
161000	11380	11380	5170	5170	3980
***X*** **_T_ (MPa)**	***X*** **_C_ (MPa)**	***Y*** **_T_ (MPa)**	***Y*** **_C_ (MPa)**	***S_L_*** **(MPa)**	***θ*** ***_i_* (^o^)**
2560	1590	73	250	113	1.5
***μ*** **_12_**	***μ***_13_	*****μ***** **_23_**	***β***	***m***	***a*** **_66_**
0.32	0.32	0.43	1059.955	0.2041	2.6
***G*** **_IC_ (N/mm)**	***G*** **_IIC_ (N/mm)**	***G*** **_kink_ (N/mm)**	***G*** **_split_ (N/mm)**		
0.26	1.002	80	80		

**Table 4:** Material properties of cohesive layers [10].

*K*_I_ (N/mm^3^)	*K*_II_ (N/mm^3^)	*σ*_Ι_^max^ (MPa)	*σ*_ΙΙ_^max^ (MPa)	*G*_IC_ (N/mm^3^)	*G*_IIC_ (N/mm^3^)	*α*
10^5^	10^5^	60	90	0.26	1.002	1

## References

[1] Hsiao H.M., Daniel I.M. (1996). Effect of fiber waviness on stiffness and strength reduction of unidirectional composites under compressive loading. Compos. Sci. Technol..

[2] Davidson P., Waas A.M. (2017). The effects of defects on the compressive response of thick carbon composites: An experimental and computational study. Compos. Struct..

[3] Davidson P., Waas A.M. (2018). Probabilistic defect analysis of fiber reinforced composites using kriging and support vector machine based surrogates. Compos. Struct..

[4] Adams D.O.H., Bell S.J. (1995). Compression strength reductions in composite laminates due to multiple-layer waviness. Compos. Sci. Technol..

[5] Adams D.O.H., Hyer M.W. (1993). Effects of layer waviness on the compression strength of thermoplastic composite laminates. J. Reinf. Plast. Compos..

[6] Wisnom M.R., Atkinson J.W. (1996). Compressive failure due to shear instability: Experimental investigation of waviness and correlation with analysis. J. Reinf. Plast. Compos..

[7] Chun H.J., Shin J.Y., Daniel I.M. (2001). Effects of material and geometric nonlinearities on the tensile and compressive behavior of composite materials with fiber waviness. Compos. Sci. Technol..

[8] Makeev A., Seon G., Lee E. (2010). Failure predictions for carbon/epoxy tape laminates with wavy plies. Compos. Sci. Technol..

[9] Davidson P., Waas A.M. (2016). Mechanics of kinking in fiber-reinforced composites under compressive loading. Math. Mech. Solids.

[10] Mukhopadhyay S., Jones M., Hallett S. (2015). Compressive failure of laminates containing an embedded wrinkle; Experimental and numerical study. Compos. Part A Appl. Sci. Manuf..

[11] Prabhakar P., Waas A.M. (2013). Interaction between kinking and splitting in the compressive failure of unidirectional fiber reinforced laminated composites. Compos. Struct..

[12] Yerramalli C.r.S., Waas A.M. (2004). The effect of fiber diameter on the compressive strength of composites—A 3D finite element based study. Comput. Model. Eng. Sci..

[13] Hasanyan A.D., Waas A.M. (2018). Compressive failure of fiber composites: A homogenized, mesh independent model. J. Appl. Mech..

[14] Sun Q., Zhou G., Meng Z., Guo H., Chen Z., Liu H., Kang H., Keten S., Su X. (2019). Failure criteria of unidirectional carbon fiber reinforced polymer composites informed by a computational micromechanics model. Compos. Sci. Technol..

[15] Yuan Y., Yao X., Niu K., Liu B., Wuyun Q. (2019). Compressive failure of fiber reinforced polymer composites by imperfection. Compos. Part A Appl. Sci. Manuf..

[16] Lemanski S.L., Wang J., Sutcliffe M.P.F., Potter K.D., Wisnom M.R. (2013). Modelling failure of composite specimens with defects under compression loading. Compos. Part A Appl. Sci. Manuf..

[17] Wang J., Potter K.D., Hazra K., Wisnom M.R. (2012). Experimental fabrication and characterization of out-of-plane fiber waviness in continuous fiber-reinforced composites. J. Compos. Mater..

[18] Wang J., Xiao Y. (2016). Some improvements on Sun–Chen’s one-parameter plasticity model for fibrous composites Part I: Constitutive modelling for tension–compression asymmetry response. J. Compos. Mater..

[19] Wang J., Xiao Y. (2016). Some improvements on Sun-Chen’s one-parameter plasticity model for fibrous composites Part II: Finite element method implementation and applications. J. Compos. Mater..

[20] Wang J., Xiao Y., Kawai M. (2019). Parameter identification problem in one-parameter plasticity model for fibrous composites. Adv. Compos. Mater..

[21] Xue K. (2018). Progressive analysis of bearing failure in pin-loaded composite laminates using an elastoplastic damage model. Mater. Sci. Appl..

[22] Xue K., Xiao Y., Wang J., Xue Y.D. (2019). Compression progressive failure analysis of unidirectional composites. Acta Mater. Compos. Sin..

[23] Chen J.F., Morozov E.V., Shankar K. (2012). A combined elastoplastic damage model for progressive failure analysis of composite materials and structures. Compos. Struct..

[24] Puck A., Schürmann H. (1998). Failure analysis of FRP laminates by means of physically based phenomenological models. Compos. Sci. Technol..

[25] Puck A., Schürmann H. (2002). Failure analysis of FRP laminates by means of physically based phenomenological models. Compos. Sci. Technol..

[26] Schultheisz C.R., Waas A.M. (1996). Compressive failure of composites part I: Testing and micromechanical theories. Prog. Aerosp. Sci..

[27] Argon A.S., Herman H. (1972). Fracture of Composites. Treatise on Materials Science and Technology.

[28] Pinho S.T., Darvizeh R., Robinson P., Schuecker C., Camanho P.P. (2012). Material and structural response of polymer-matrix fibre-reinforced composites. J. Compos. Mater..

[29] Matzenmiller A., Lubliner J., Taylor R.L. (1995). A constitutive model for anisotropic damage in fiber-composites. Mech. Mater..

[30] Sun C.T., Chen J.L. (1989). A simple flow rule for characterizing nonlinear behavior of fiber composites. J. Compos. Mater..

[31] Puck A., Kopp J., Knops M. (2002). Guidelines for the determination of the parameters in Puck’s action plane strength criterion. Compos. Sci. Technol..

[32] Pinho S.T., Iannucci L., Robinson P. (2006). Physically-based failure models and criteria for laminated fibre-reinforced composites with emphasis on fibre kinking: Part I: Development. Compos. Part A Appl. Sci. Manuf..

[33] Reinoso J., Catalanotti G., Blázquez A., Areias P., Camanho P.P., París F. (2017). A consistent anisotropic damage model for laminated fiber-reinforced composites using the 3D-version of the Puck failure criterion. Int. J. Solids Struct..

[34] Jiang W.G., Hallett S.R., Green B.G., Wisnom M.R. (2007). A concise interface constitutive law for analysis of delamination and splitting in composite materials and its application to scaled notched tensile specimens. Int. J. Numer. Methods Eng..

[35] Mi Y., Crisfield M.A., Davies G.A.O., Hellweg H.B. (1998). Progressive delamination using interface elements. J. Compos. Mater..

[36] Knops M. (2008). Analysis of Failure in Fiber Polymer Laminates: The Theory of Alfred Puck.

[37] Wiegand J., Petrinic N., Elliott B. (2008). An algorithm for determination of the fracture angle for the three-dimensional Puck matrix failure criterion for UD composites. Compos. Sci. Technol..

[38] Schirmaier F.J., Weiland J., Kärger L., Henning F. (2014). A new efficient and reliable algorithm to determine the fracture angle for Puck’s 3D matrix failure criterion for UD composites. Compos. Sci. Technol..

[39] Hashin Z. (1980). Failure criteria for unidirectional fiber composites. J. Appl. Mech..

[40] Inc A. (2016). ABAQUS/Explicit, 2016.

[41] Xie N., Smith R.A., Mukhopadhyay S., Hallett S.R. Modelling the mechanical properties of wrinkled composites from NDT data. Proceedings of the 20th International Conference on Composite Materials.

[42] Xie N., Smith R.A., Mukhopadhyay S., Hallett S.R. (2018). A numerical study on the influence of composite wrinkle defect geometry on compressive strength. Mater. Des..

